# The direct anterior approach to the hip: a useful tool in experienced hands or just another approach?

**DOI:** 10.1186/s42836-021-00104-5

**Published:** 2022-01-02

**Authors:** John Realyvasquez, Vivek Singh, Akash K. Shah, Dionisio Ortiz, Joseph X. Robin, Andrew Brash, Mark Kurapatti, Roy I. Davidovitch, Ran Schwarzkopf

**Affiliations:** Department of Orthopedic Surgery, Division of Adult Reconstruction, NYU Langone Health, 301 East 17th Street, New York, NY 10003 USA

**Keywords:** Direct anterior approach, Total hip arthroplasty, Hip replacement

## Abstract

The direct anterior approach (DAA) to the hip was initially described in the nineteenth century and has been used sporadically for total hip arthroplasty (THA). However, recent increased interest in tissue-sparing and small incision arthroplasty has given rise to a sharp increase in the utilization of the DAA. Although some previous studies claimed that this approach results in less muscle damage and pain as well as rapid recovery, a paucity in the literature exists to conclusively support these claims. While the DAA may be comparable to other THA approaches, no evidence to date shows improved long-term outcomes for patients compared to other surgical approaches for THA. However, the advent of new surgical instruments and tables designed specifically for use with the DAA has made the approach more feasible for surgeons. In addition, the capacity to utilize fluoroscopy intraoperatively for component positioning is a valuable asset to the approach and can be of particular benefit for surgeons during their learning curve. An understanding of its limitations and challenges is vital for the safe employment of this technique. This review summarizes the pearls and pitfalls of the DAA for THA in order to improve the understanding of this surgical technique for hip replacement surgeons.

## Introduction

The direct anterior approach (DAA) for total hip arthroplasty (THA) was first described by Carl Heuter in the late 1800s and subsequently augmented by Smith-Peterson, Light and Keggi, and the Judets [1–4]. Modern-day literature frequently refers to this surgical method interchangeably as both the Hueter and Smith-Petersen approach when identifying the anterior-based incision that utilizes the interval to the hip joint through the tensor fasciae latae (TFL) and the sartorius muscles [1]. Several proposed advantages of the DAA, alongside the desire to perform hip reconstruction through a smaller incision and tissue-sparing methods, have led to the newfound popularity and utilization of the DAA in primary THA over the past decade [5]. While many orthopedic surgeons consider the appropriate use of the DAA exclusively for primary joint replacement, several investigations have noted its utility for complex revision procedures and hip fractures [6–10]. Advocates of the DAA have cited decreased pain, length of stay (LOS), dislocation rate, and expedited recovery as a rationale for employing the DAA [11].

At the annually held 2018 American Association of Hip and Knee Surgeons (AAHKS) meeting, members were polled as to their preference regarding surgical technique for THA [9]. The results of the survey showed that 56% of respondents reported using the DAA in their practice [9]. Furthermore, an audience survey conducted by Abdel et al [12] demonstrated similar trends as 40% of surgeons polled at the same meeting stated that they employed the DAA during primary THA [12]. AAHKS members who chose not to employ the DAA stated that their decision was due to perceived worse outcomes, no significant clinical benefits compared to other surgical approaches, and an inherently steep learning curve that may lead to increased complication rates [13–15]. Among the members who chose to utilize the DAA in their practice, some stated that they also employ the posterior approach, but solely in a select subset of cases such as complex revisions, patients with specific body habitus types, complex anatomy, or both. This review aims to summarize the published literature on the direct anterior approach to THA with a focus on comparative key pearls and pitfalls. An understanding of the surgical technique and its outcomes can ultimately help surgeons better evaluate the role of the DAA in contemporary hip arthroplasty.

### Utility and challenges

As with more traditional surgical approaches for THA, certain patient considerations make this approach challenging. While a few previous studies have recommended using the DAA in patients of nearly all body habitus and hip conditions [16, 17], the ideal candidate has been described as a flexible, non-muscular patient with valgus femoral neck and good femoral offset [18]. Therefore, it is most appropriate to employ this surgical approach in slender patients with a body mass index (BMI) < 30 kg/m^2^ [19]. Although obesity can make any THA approach difficult, subcutaneous fat in the anterior hip region tends to be minimal compared with other aspects of the hip (posterior and lateral). However, patients with a large abdominal panniculus, particularly those with tissue that overlaps the upper thigh, present an additional challenge when using the DAA. This overlapping tissue has the potential to create a moist environment that may result in chronic skin irritation or fungal infection making these patients more prone to wound complications. Therefore, these individuals require additional vigilance to ensure proper healing of the skin incision [5].

Additionally, some anatomic variability of the native hip and pelvis may make the DAA more difficult to perform. A wide or horizontal iliac wing limits access to the femoral canal for broaching and femoral component placement. Acetabular protrusio positions the femoral canal adjacent to the pelvis and obstructs access to the femur. A neck-shaft angle with decreased offset positions the femoral canal deeper in the thigh, and anatomy associated with obese muscular males limits the space available for adequate component placement [20]. The anterior exposure may be unsuitable if the patient has a deficient posterior acetabular wall from previous hardware or trauma, or if posterior acetabular augmentation is being considered [21]. Additionally, previous literature has described utilizing the posterior approach in place of the DAA for retained hardware secondary to a previous acetabular fracture or if the posterior column is retained during conversion THA [22].

### Contraindications to the DAA

As with all things, it is important to know when to employ a technique and when to reassess the value of a particular approach for surgical management. Although the DAA has many potential advantages there is still a subset of patients who may not necessarily be the best candidates for this surgical approach. Sang et al [23], recently reviewed the effect of BMI and hip anatomy on the DAA. They found that on average patients with a higher BMI had longer operative time, increased intraoperative bleeding, and a higher rate of complications than the cohort with a lower BMI [23]. Some of their noted complications were intraoperative femur fracture, damage to the lateral femoral cutaneous nerve (LFCN), and hematoma formation at the wound site. In their analysis of the anatomical differences that may influence outcomes in the DAA, they found that patients with a GT/ASIS ratio of greater than 1.17 had significantly shorter operative times and lower amounts of intraoperative bleeding compared to patients with a GT/ASIS ratio of < 1.17. The GT/ASIS ratio represents the difference in length between two parallel lines. One is drawn horizontally through the lateral borders of each GT and another parallel line that connects both ASIS respectively. The ratio is the resulting length difference in the GT/ASIS. Therefore, this may represent patients with a higher BMI and increased anatomical variations may be relatively contraindicated in receiving a THA utilizing the DAA [23].

Similarly, in a case-control study of 651 consecutive DAA arthroplasties over 3 years, Jahng et al [24] found that BMI was significantly and independently associated with wound complications and necessary reoperation. Furthermore, their analysis demonstrated that diabetes mellitus had an increased odds ratio for developing wound complications following DAA THA. This recapitulates that a high BMI maybe is a relative contraindication to DAA and that diabetes mellitus may represent the second cohort of patients in whom DAA may not be the ideal approach [24].

Sali et al [25] recently examined the relationship between BMI, age at the time of operation, and difference in preoperative and postoperative hemoglobin and their effects on postoperative complications and readmission rates [25]. Their findings indicate that patients with age > 60 at the time of operation had more postoperative complications than younger patients. Furthermore, patients with > 3 comorbidities were at higher risk for medical complications following DAA THA than those with < 3 comorbidities. Furthermore, patients with a BMI > 30 had a higher risk of 30-day readmission than those with a lower BMI. Finally, a difference of > 3 between preoperative and postoperative hemoglobin had a higher risk of 30- and 90-day readmission following DAA THA. This indicates that patients with more existing comorbidities, a higher BMI, or major difference between preoperative and postoperative hemoglobin may have a relative contraindication to undergoing THA through the DAA technique [25].

### Surgical technique

#### Patient positioning, incision, and superficial dissection

The patient is placed supine on a radiolucent table or traction table. If a traction table is used, it is paramount to inspect that boots are locked in place and all traction is off [26] and the peroneal post is well padded to avoid peroneal nerve neuropraxia [27–29]. If a radiolucent operating room table is used, the patient should be positioned with the hip located over the table break; this allows for hyperextension of the hip joint during the procedure [26]. Obese patients should have their pannus retracted with adhesive tape to avoid interference with exposure [17]. A Mayo stand should be placed alongside to allow for lower limb figure-four adduction during the femoral exposure [26].

**Fig. 1:**
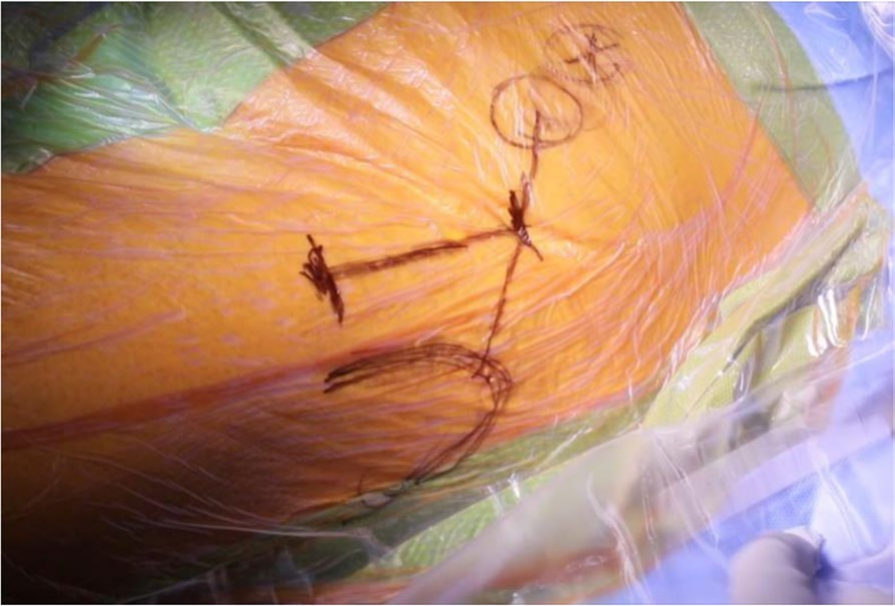
Incision guide

**Fig. 2:**
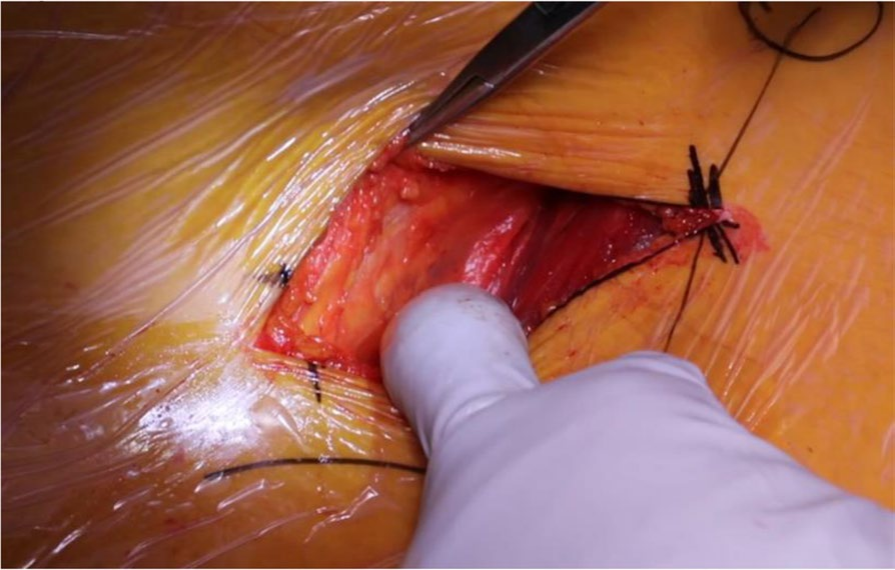
Medial leaf of the fascia and blunt dissection

**Fig. 3:**
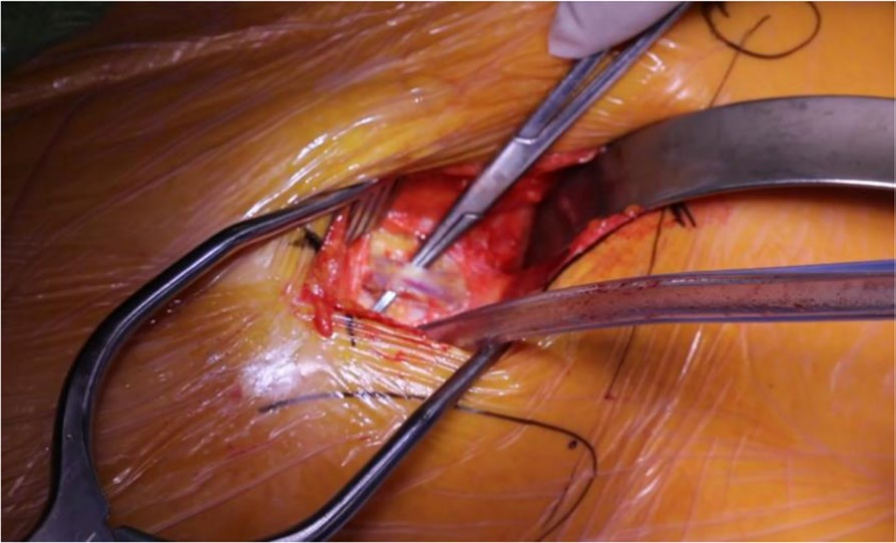
Perforating vessels of the lateral circumflex artery and vein

The incision is marked out based on the Smith-Petersen approach by palpating the anterior superior iliac spine (ASIS) and the tip of the greater trochanter (GT) [30]. The initial incision is approximately 7-10 cm in length and should begin approximately 2-3 cm lateral and 2-3 cm distal to the ASIS and extend toward the lateral femoral condyle and the fibular head (Fig. 1) [26, 30]. The space between the sartorius and TFL should be identified. Blunt dissection may be utilized under the medial fascia as the interval is developed between the sartorius and the TFL (Fig. 2) [26]. Care should be taken not to damage the lateral femoral cutaneous nerve during dissection of the subcutaneous fat. The fascia is then sharply incised, with the medial portion carefully peeled off the muscle (Fig. 2). A blunt cobra retractor is placed superior to the lateral capsule to retract the abductors and a second large Hohmann retractor is placed inferior to the femoral neck [26, 30]. A cerebellar retractor or Morse retractors are placed distally, separating gluteus medius laterally and rectus femoris medially. This exposes the ascending branch of the lateral femoral circumflex artery over the intertrochanteric line (Fig. 3) [26]. Care should be exercised to cauterize this vessel and its concomitant veins, as significant bleeding will be encountered if this is not performed [26, 30].

#### Deep dissection to the anterior capsule

**Fig. 4:**
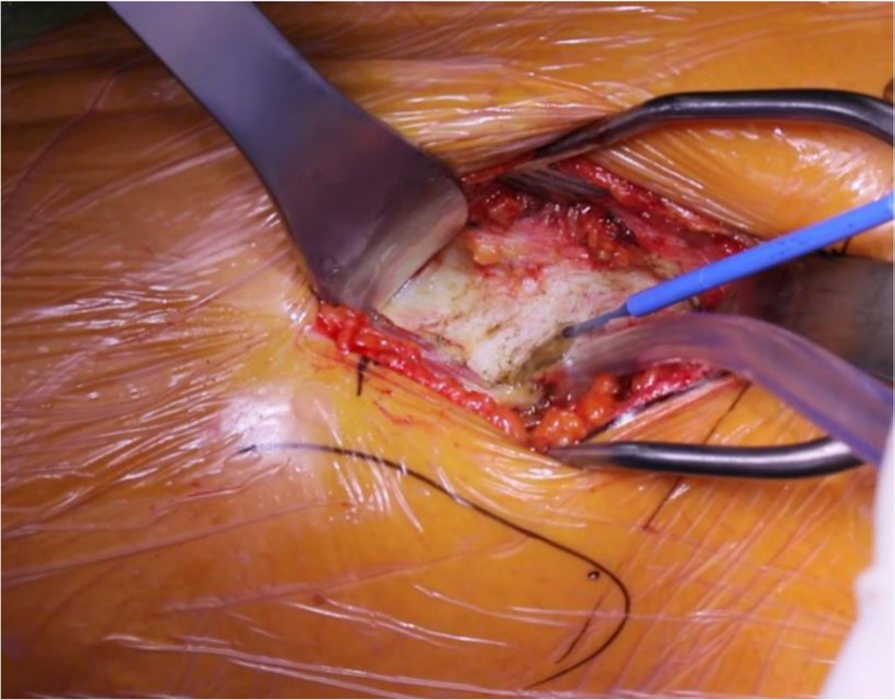
Homman retractor inserted medially between the anterior fat pad and the capsule with commencement of the capsulotomy

**Fig. 5:**
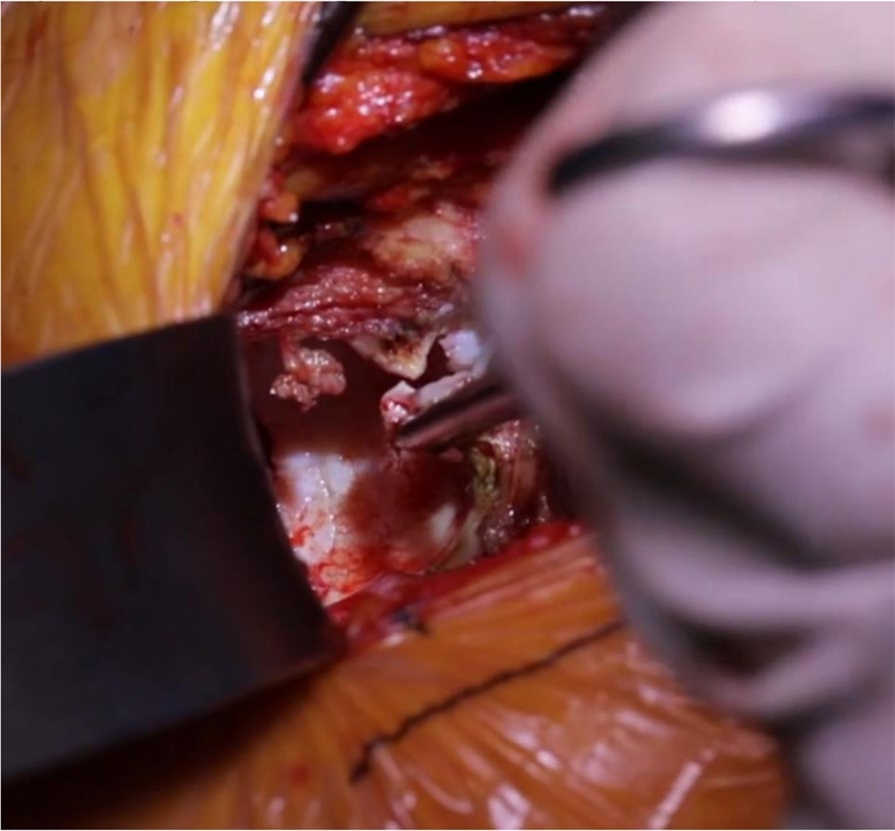
Superior capsulectomy and labrectomy

Incised next is the deep fascia, which overlies the precapsular fat (Fig. 4). Once the capsule is exposed, a second cobra retractor is placed below the inferior femoral neck, anterior to vastus lateralis, with the handle directed towards the contralateral knee [26]. Additionally, it has been noted that a third curved retractor can be helpful to elevate the rectus tendon proximally in patients with larger body habitus [26]. Next, an anterior capsulotomy or capsulectomy is carried out depending on surgeon preference (Fig. 5). The anterior capsule can undergo partial or complete excision or be incised and tagged for later repair. Recently, Vandeputte et al [31] conducted a randomized controlled trial comparing capsulectomy versus capsulotomy with the DAA. Their group found no clinical difference between preservation and resection of the capsule during primary THA. However, they did advise that during training, it may be advantageous to perform a capsulectomy to increase visibility for a surgeon new to the technique [31]. It is beneficial to begin on the inferior neck and work distal to proximal onto the capsule. In addition, the hip can be flexed 20 degrees to release tension off the rectus if needed. It is paramount to ensure you are on the capsule and that no muscle is under the retractor as the neurovascular bundle lies on the other side of the rectus muscle. An inverted T capsulotomy or “L” capsulotomy can be performed along the intertrochanteric line and proximally up the femoral neck [26, 30]. Studies have shown no difference in infection or instability rates between capsulotomy or capsulectomy [32].

#### Femoral neck osteotomy and acetabular exposure

Following the removal of the capsule, fluoroscopy should be utilized to measure the femoral neck osteotomy. For large heads or those with numerous osteophytes, a napkin ring osteotomy may be pertinent [30]. A corkscrew is then inserted through the cortical side of the femoral head or the femoral neck cut, and the head is then pulled out while ensuring not to damage the TFL [26]. If there is concern about damaging the TFL, surgeons may choose to employ a technique recently described by Zhao et al [33], in which the anterior joint capsule can be flipped over TFL and sutured to the skin to protect the muscle. This may be beneficial prior to the removal of the head and reaming the acetabulum.

Once the head and neck are removed, gross traction is placed on the operative limb to help with visualization of the acetabulum. The labrum is then excised, the bony anatomy of the socket is assessed, and acetabular reaming is performed under fluoroscopy [30]. The acetabular cup is then inserted.

#### Acetabular preparation and Acetabular component implantation

To determine the cup position, several tools can be utilized, including intraoperative navigation, computer software based on C-arm imaging, or C-arm techniques [34]. Cup anteversion can be more difficult to quantify than inclination and plays a significant role in preventing instability in patients with hip-spine pathology [35, 36]. At our institution, we employ a technique described by Boettner et al [37] that correlates C-arm rotation with cup anteversion.

First, an AP pelvis X-ray is obtained, which mirrors the standing preoperative AP pelvis radiograph. The C-arm is directed toward the operative hip to obtain an AP hip view. An assistant may measure the inclination of the cup on the C-arm monitor using a protractor, or the surgeon can estimate based on his or her experience. The C-arm is then tilted away from the operative side until it is in plane with the cup and a straight line is visualized at the cup’s edge. The degree of C-arm tilt is recorded and converted to cup anteversion. For standard 40/20 or 40/15 positioning, C-arm tilt of approximately 25-30 degrees is recommended.

Once the cup is implanted and its position verified, all traction is now released from the femur. Next, the leg is placed into 90-120 degrees of external rotation based on patient anatomy and soft tissue tension. The femoral releases are then performed.

#### Femoral exposure

**Fig. 6:**
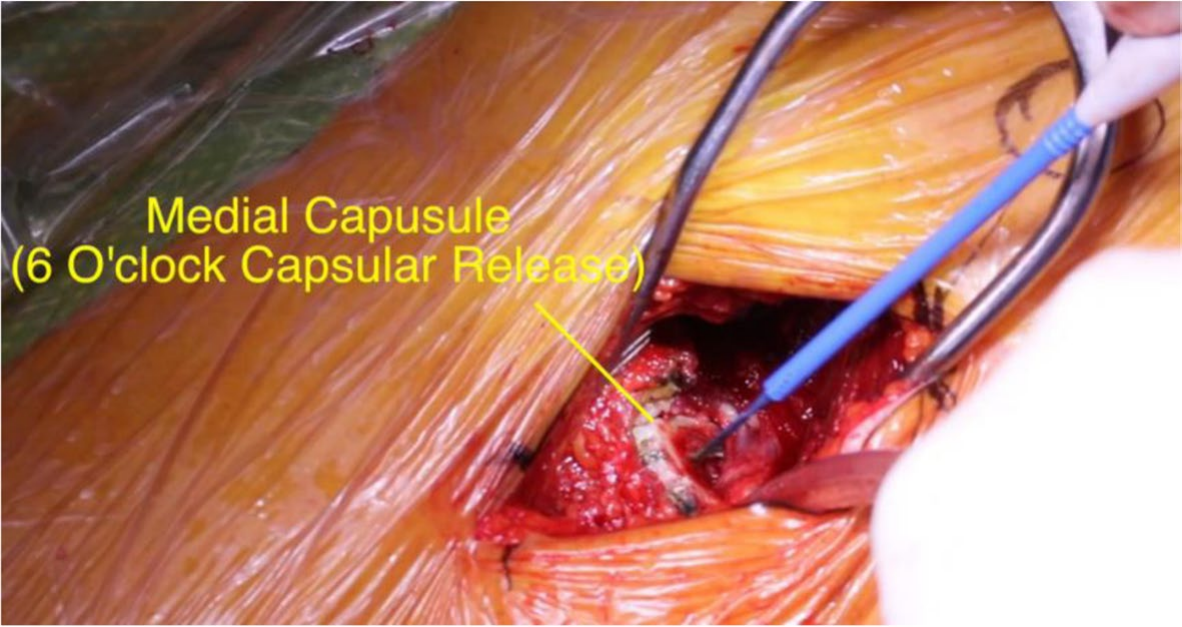
Medial capsular release. Thickening of the pubofemoral ligament

The first release is the 6 o’clock release which releases the tight pubofemoral ligament (Fig. 6). This is carried down to the level of the lesser trochanter without violating the iliopsoas tendon. The leg is then carefully lowered towards the floor and adducted until the proximal femur comes into view. A sharp wide Hohmann retractor is placed over the superior neck.

**Fig. 7:**
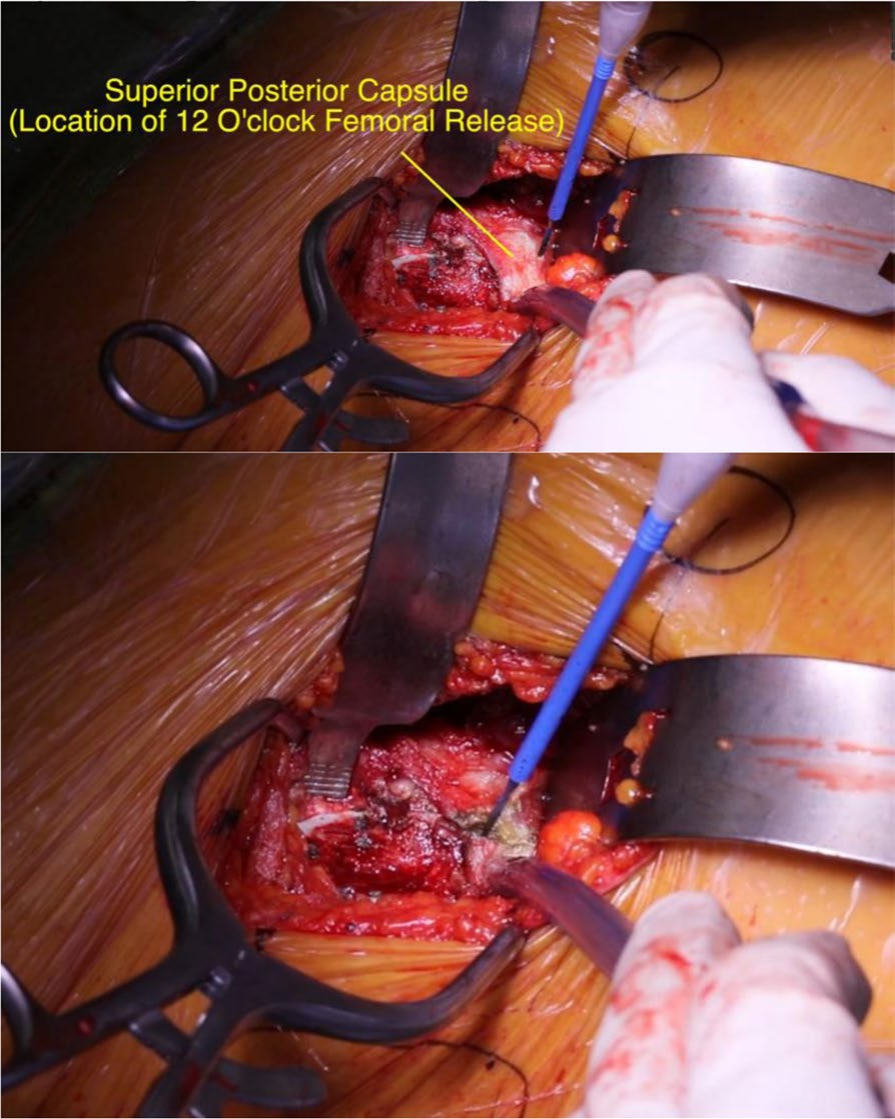
Superior posterior capsular release

The second release is the 12 o’clock release, which opens the superior capsule to allow the femur to be translated ventrally towards the surgeon (Fig. 7). If done correctly, the femur can be translated several centimeters. If the femur does not move much or a direct view into the femur with the broaches is unattainable, it is recommended the femur be reset by bringing the leg back into neutral and start the releases again. This may aid in releasing any soft tissue that is interfering with the translation of the femur.

**Fig. 8:**
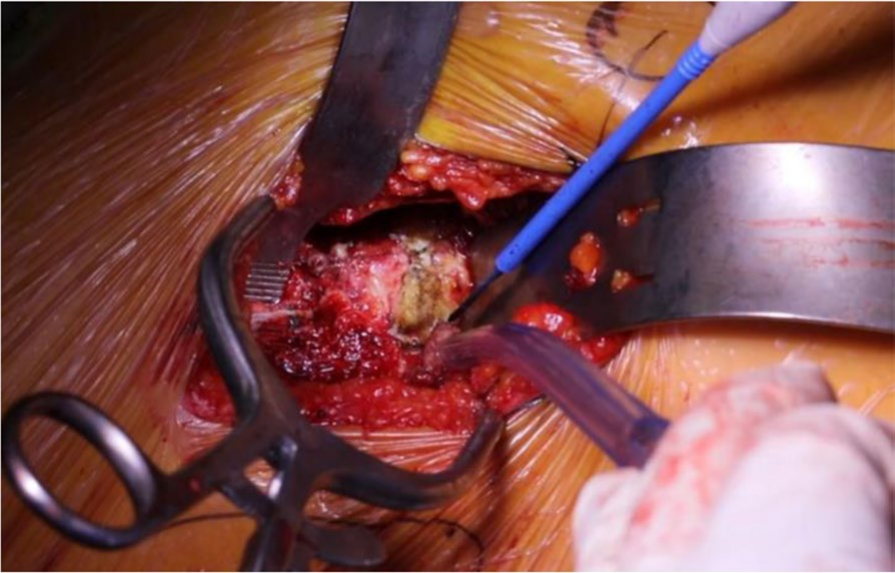
Elevation and visualization of the proximal femur without the necessity of a femoral bone hook

If performed optimally, excellent exposure of the proximal femur can be obtained without the use of a femoral elevator (Fig. 8). With knowledge of the surrounding anatomy and accurate retractor placement, this technique is powerful and reproducible.

#### Femoral preparation, trialing and component implantation

**Fig. 9:**
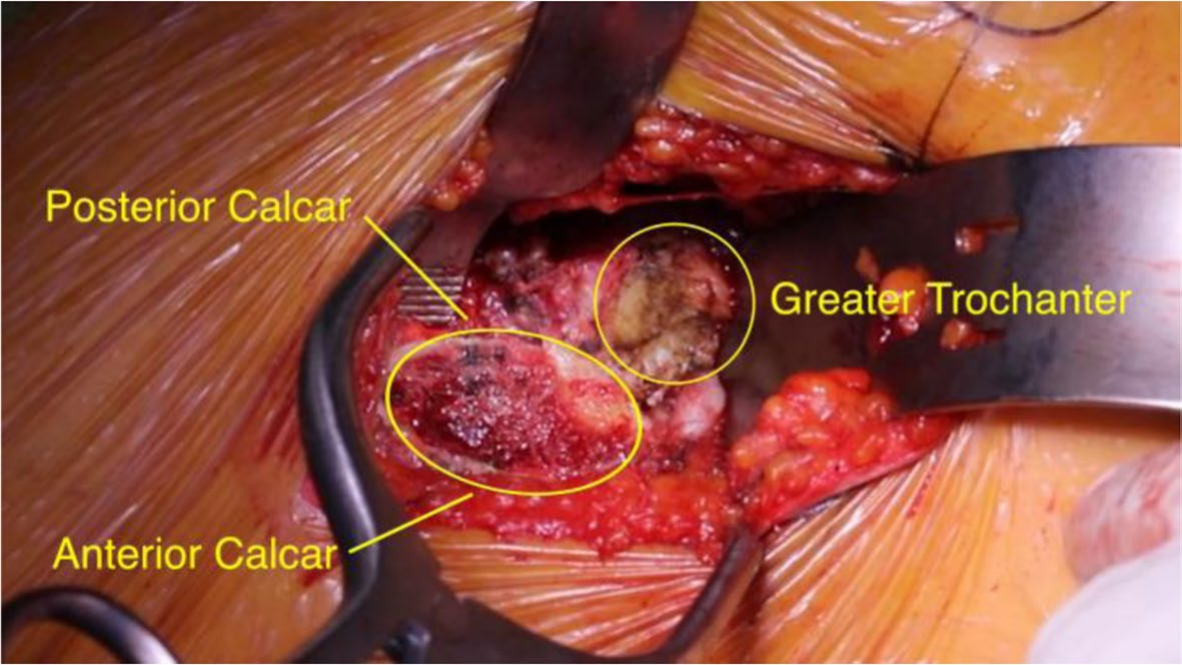
Anatomical view of the calcars and greater Trochanter

While broaching the femur, care must be taken to avoid excessive anteverting as this may lead to increased rates of postoperative dislocation. One must ensure that appropriate lateralization is employed to maintain an adequate fit (Fig. 9). A trial implant should then be placed in accordance with preoperative planning. The position of the implant should be confirmed with a C-arm, and leg lengths should be evaluated using the lesser trochanters to ensure no leg length discrepancy exists. The trial implant should then be dislocated; the surgeon may need to employ a bone hook to necessitate the dislocation. The leg is then externally rotated to approximately 130 degrees and placed in extension and adduction to expose the femur before final implant placement.

**Fig. 10:**
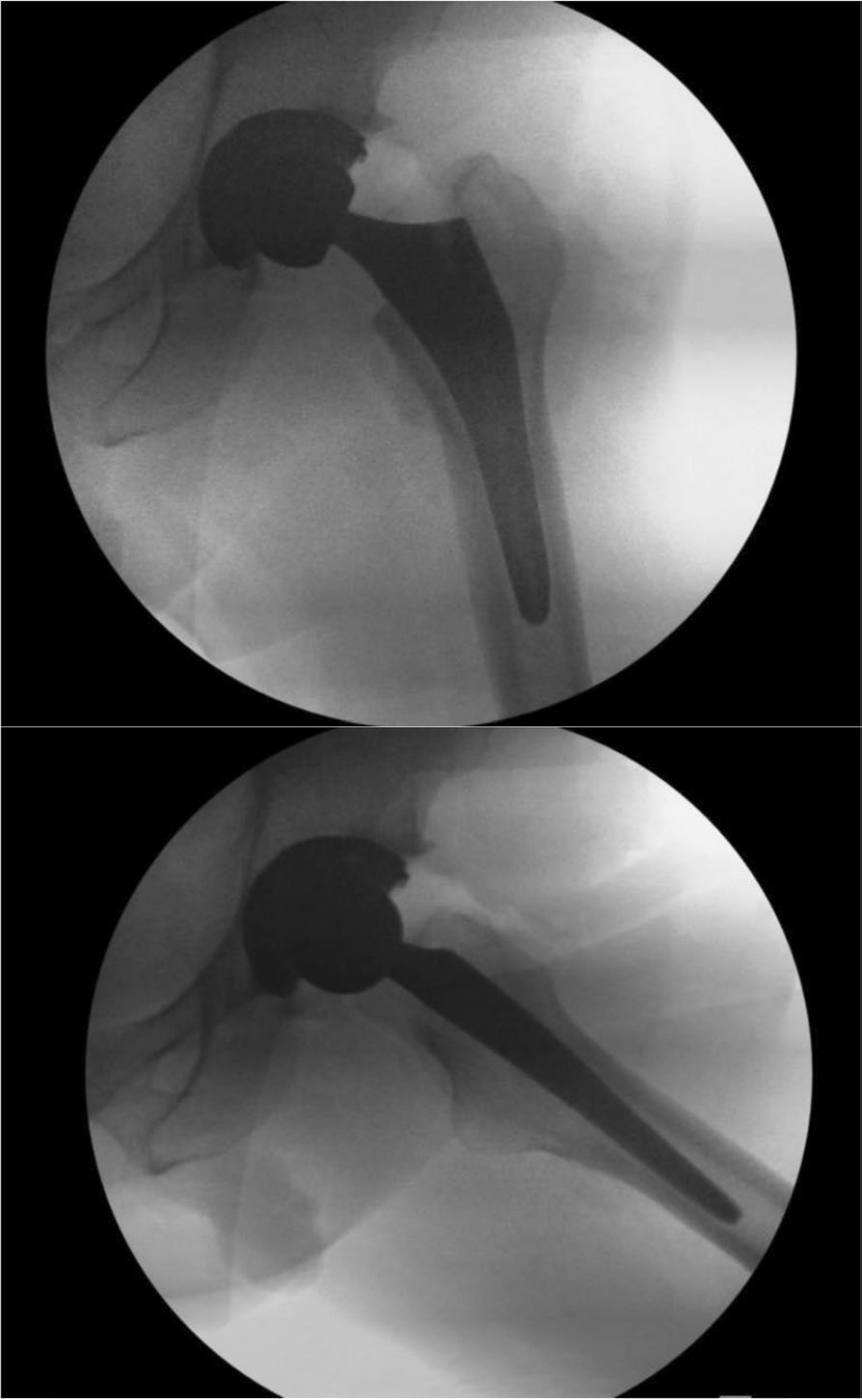
Final fluoroscopy demonstrating proper implant placement

During the final placement, it is paramount to assess the broach to ensure there is no subsidence and that stability can be maintained. Implant adjustment may be assessed and modified through the femoral head component. If the surgeon is confident in the trial measurement, the final implant can be placed (Fig. 10).

#### Wound closure

Once the final implants are in, stability testing is performed and the wound is thoroughly irrigated using chlorhexidine lavage, closure of the wound should begin. Commence with a capsular repair if necessary, followed by the closure of the TFL fascia through either a running or interrupted suture [26]. Drain placement is dependent on surgeon preference. However, it should be noted that the placement of a drain may better assist the surgeon in estimating blood loss post-THA [38, 39].

### Learning curve

The DAA for THA is a highly popularized and marketed approach. However, as with any surgical technique, it is not without an associated learning curve. There is also a question as to whether the utilization of this approach provides superior clinical outcomes compared to other approaches. As such, it is necessary to examine this approach and determine whether it should be heralded as innovative or simply described as another surgical approach for THA.

The learning curve associated with the DAA has been evaluated by multiple authors to determine the number of cases one must perform to become sufficient and comfortable with the technique. Nairn et al [40] performed a meta-analysis analyzing the mean operative time for surgeons when employing the DAA. They found that mean operative time by case 100 was significantly shorter than case one and that the complication rate decreased significantly in later groups as the surgeon conducted more cases. Furthermore, their study indicated that mean operative time began to plateau at case 100. This indicates that a younger or inexperienced surgeon may have to perform roughly 100 cases before they can demonstrate mastery of the technique. A prospective study conducted by Pirruccio et al [41] examined the operative results of a single surgeon’s first 100 cases using the DAA and the last 100 consecutive posterior approach THA cases after 7 years in practice. Their results demonstrated that there was no significant difference in complication rate, estimated blood loss (EBL), or morbidity rate when using the DAA compared to the posterior approach. Additionally, in their recommendations, the authors suggested a systematic method to train new surgeons in the technique. They estimated that, by case 60, the “learning” surgeon could be cleared by the “experienced” surgeon to be an instructor in the technique, if they have demonstrated significant mastery in the approach. This indicates that under proper guidance, a surgeon could develop mastery of the technique by case 60. They concluded that the DAA may be implemented without any significant adverse risk when a structured learning process is maintained and performed.

Multiple studies have examined the learning curve and found that with proper training and exposure to a preset number of cases, the DAA can be implemented with minimal complication rates [9, 13, 42–44]. However, it should be noted that the majority of these studies described mentorship or senior surgeon instruction for those new to the approach.

### Perceived advantages of the DAA

Given the increasing popularity of the DAA, it is prudent to review the advantages, perceived or otherwise, this exposure offers compared to other approaches. Proponents of the DAA approach reference a litany of advantages when advocating for the DAA. We will review short**-** and long-term recovery, pain, in-hospital length of stay, and dislocation risk to review the perceived advantages of the DAA compared to other approaches.

### Recovery

Proponents of the DAA argue that the DAA approach provides a speedier recovery for patients than other approaches. However, there are conflicting data to support this statement. Firstly, recovery is a blanket term that can encompass multiple areas of the patient’s postoperative course. It can be extrapolated from a patient’s in-hospital LOS, whether an assistive device is required following the procedure and for how long, the length of time until return to work, or return to sports and leisurely activities. In a randomized controlled trial of 60 patients, Zhao et al [45] compared the DAA to the posterolateral (PL) approach to assess differences in EBL, LOS, and Patient Reported Outcomes (PRO) scores. They found that patients who underwent the DAA had greater Harris Hip Scores (HHS) and University of California Los Angeles Activity Scores at 3 months. However, at 6 months, the differences in PRO scores between groups were similar. Peters et al [46] examined the PRO scores of 12,274 patients from the Dutch arthroplasty registry, aiming to compare the DAA to the PL approach. They found that, at 3 months, patients who underwent the PL approach and DAA had similar PRO scores. This raises some doubt as to the validity of the superior recovery provided by the DAA.

Although Tauton et al [47] provided evidence that patients who underwent DAA THA discontinued walking aids sooner, the functional recovery scores cast doubt as to whether this difference is beneficial or yields superior clinical outcomes. In a previous study performed at our institution, Singh et al [48] compared the DAA to the posterior approach using the Forgotten Joint Scores (FJS). Although our initial data suggest that patients who underwent the DAA achieved higher FJS at 12 weeks, we found that this difference dissipated when the data were normalized to account for surgeon experience with their respective surgical approaches. Although long-term differences between surgical approaches are relatively similar, the primary reason some surgeons utilize the DAA is to improve the short-term recovery and to focus on the patient experience during the THA procedure and episode of care.

Furthermore, two studies are of interest regarding outcomes and risk of dislocation. Martusiewicz et al [49] recently compared PRO scores and functional outcomes between the DAA and posterior approach. Their data indicate that patients who underwent the DAA had improved modified Harris Hip Scores (mHHS) at 5 weeks postoperatively, discontinued their walking aids 8 days earlier, and drove cars 5 days earlier than their counterparts who underwent primary THA through the posterior approach [49]. In addition to this data, a recent review of the Kaiser Permanente Joint Registry by Charney et al [50], indicates that patients who underwent primary THA via the DAA were at lower risk for dislocation, and lower risk of revision for instability or periprosthetic fracture, and had lower readmission rates than their counterparts who underwent THA through the posterior approach [50].

### Pain

Pain is another measurement that advocates of the DAA present as evidence for its utilization. Once again, pain is an ambiguous and convoluted term that encompasses multiple areas and categories. There may be institutional differences in pain protocols and anesthetic usage that can account for differences in pain experienced by patients. Furthermore, periarticular and wound anesthetic cocktails may differ between institutions and may act as confounders for measurements of pain when comparing one approach to another. Lastly, pain is a subjective measure that is difficult to quantify and standardize across groups and populations. Zhao et al [45] compared postoperative pain using self-reported pain scales in patients who underwent the DAA and PL approaches. They demonstrated that patients who underwent the DAA THA reported lower pain at twenty-four, forty-eight, and seventy-two hours compared to those who underwent PL approach THA. However, the question of whether these differences are clinically significant remains.

Cheng et al [51] compared the Western Ontario and McMaster Universities Arthritis Index (WOMAC) scores for pain and stiffness in seventy-two patients and found no statistical difference at two, six, and twelve-week intervals between patients who underwent the DAA or posterior approach [51]. In a multicenter propensity score-matched study comparing the DAA to the PL approach, Sauder et al [52] found no evidence for superior PRO scores in patients treated with the DAA. Patients treated with the DAA were less likely to achieve a Patient Acceptable Symptomatic State (PASS) for the Harris Hip Score and Hip Outcome at their postoperative visit than patients treated with the PL approach [52]. They also found no statistical difference between the DAA cohort and PL cohort at 1 year in the minimal clinically important difference (MCID) for pain.

### In-hospital length of stay (LOS)

LOS is a vital measure not only for patient safety but also fiscally. Decreases in LOS can lower hospital-acquired infections, decrease costs, and allow for an earlier return to work or play, which in turn may result in increased patient satisfaction and outcomes. Therefore, any procedure or advance in treatment that may shorten LOS is a highly valuable commodity. However, LOS is dependent on factors that are out of the surgeon’s control, making it highly variable, such as preexisting conditions, age, gender, smoking status, etc. In addition, there are procedure-related factors that may affect LOS, such as operative time, start time, and approach. Cheng et al [51] examined the LOS and time to home discharge between the DAA and posterior approach THA and found no significant difference between groups [51]. In a meta-analysis of seventeen studies, Higgins et al [15] found that there was a significant decrease in LOS when using the DAA compared to the posterior approach. Although, their consensus was that the current level of clinical evidence demonstrates no clear superiority of one approach to the other [15]. Ultimately, they recommended that the choice of surgical approach should be determined by the surgeon’s knowledge of and experience with the technique, patient characteristics, and both surgeon and patient preferences.

### Dislocation risk

There is an assertion that the DAA may decrease the rate of postoperative dislocations. However, one must examine the claims made for the DAA versus the reality of the clinical data. Trousdale et al [53] surveyed 166 patients about the DAA. Their data indicated that the majority of information relayed regarding the DAA came from family and friends. Thus, patient perceptions are not in accordance with the published data but rather influenced by family, friends, and current marketing of the technique. In a propensity score-matched study, Maratt et al [54] analyzed the rate of dislocation between the DAA and posterior approach THA. They found no significant difference in dislocation rates between both cohorts [54]. However, they did report that the posterior approach was associated with longer LOS, increased fracture risk, increased blood loss, and postoperative hematoma formation. Similarly, Huerfano et al [55], found no significant difference in dislocation rates between DAA and PL in their meta-analysis of 25 studies containing 7172 patients. In contrast, Siljander et al [56], while examining 5341 THA procedures (3162 PL, 1846 DAA, and 333 Direct Superior), found that the dislocation rate in DAA was lower when compared to the other cohorts. However, this finding failed to reach statistical significance. Charney et al [50] examined a large dataset of 38,399 patients and showed that patients treated with the DAA had lower rates of dislocations and fewer revisions for instability compared to the posterior approach [50]. However, it should be noted that**,** of the 38,399 primary THA cases included in this study, only 6428 (16.7%) were performed using the DAA. Conversely, Pincus et al [57] found that patients treated with the DAA were at increased risk of dislocation when compared to other approaches [57]. However, this study consisted of population-level data and did not control for the surgeon or hospital volume and experience with the DAA.

The risk of dislocation is indeed a serious consideration for the surgeon when deciding which approach to employ for primary THA. It has been noted that both the DAA and lateral approaches have lower rates of dislocation compared to the posterior approach. Numerous reports presented the dislocation rate of the DAA. In a review of 494 cases utilizing the DAA, Matta et al [58] reported that the rate of dislocation for the DAA was 0.61% [58]. Sariali et al [59] reported a dislocation rate of 1.5% in patients undergoing the DAA [59]. In addition, Siguier et al [60] reported the dislocation rate to be as low as 0.96% in their review of 1037 cases of primary THA that utilized the DAA [60].

Rates of dislocation for the posterior approach are significantly higher than the DAA. Tsukada et al [61] recently compared the dislocation rates between the DAA and posterior approach. In their review of 316 cases, they found the dislocation rate in patients who underwent the posterior approach to be 4%, which was significantly higher than in patients who underwent the DAA.

The lateral approach has decreased dislocation rates when compared to the posterior approach. Demos et al [62], reported the dislocation rates of 0.4% for patients undergoing primary THA through the direct lateral approach. Furthermore, Masonis et al [63] reviewed 3484 cases of primary THA that utilized the direct lateral approach. They reported a dislocation rate of 0.55%. Therefore, one can see that the rates of dislocation for the DAA or direct lateral are notably lower than the posterior approach for primary THA.

### Nerve injury

Nerve injury during THA is an unfortunate complication that can occur during primary THA. The neurological structures that are at risk depend greatly on the approach selected, the surgeon’s confidence in the approach, and a thorough knowledge of anatomy during exposure and implantation. Several mechanisms can cause nerve injury during THA, including thermal injury, direct trauma, compression injuries during instrument placement or manipulation, stretch injury during leg-lengthening, and injuries due to component positioning [64].

The major nerve that is at risk of injury during the DAA is the LFCN. It has been noted that the LFCN has a variable course around the ASIS and passes through the subcutaneous tissue between the sartorius and TFL [64]. Some estimates place the injury of the LFCN as high as 80% when utilizing the DAA. It is advisable, when developing the internervous plane, to use careful blunt dissection when developing the plane between the sartorius and TFL to prevent neuropraxia or neurolysis. Though the incidence of injury to the LFCN can be quite high, injury usually resolves without any long-term sequelae.

With the posterior approach, the structure at risk for injury is the sciatic nerve, more specifically, the common peroneal branch. Some studies have noted that the incidence of sciatic nerve injury during the posterior approach is as high as 1.3%. Given the large distribution of the sciatic nerve, injury to this area can have devastating lifelong consequences. Farell et al [65] examined motor nerve injuries of 27,000 patients following THA. Of the fourteen sciatic nerve injuries identified, nine of these had partial or no recovery after a follow-up of 6 years.

The direct lateral approach carries with it a risk of injury to the superior gluteal or femoral nerve. Due to its path passing through the gluteus medius and minimus, approximately 5 cm proximal to the GT, the superior gluteal nerve is at significant risk during the direct lateral approach [64]. Injury to this nerve can lead to a Trendelenburg gait secondary to abductor insufficiency. Additionally, the femoral nerve is responsible for the majority of hip flexion and knee extension in the lower extremity. It has been noted that hematoma formation and tethering around the Poupart ligament can lead to femoral nerve injury following THA [65].

Injury to the nervous structures during each approach carries with them their own set of challenges that the surgeon must be aware of prior to choosing their approach. Furthermore, one must be aware that signs of nerve injury have been reported to appear more than 24 hours post-THA. Farrell et al [65], in their review of 27,000 patients, states that twenty-one out of their forty-seven identified nerve palsies were diagnosed two to 7 days following THA.

### Intraoperative fracture risk

Intraoperative fractures are catastrophic complications that can be encountered during primary THA. Along with increasing the functional recovery time for the patient, fractures can cause difficulty in weight-bearing postoperatively, increase surgical time, and can lower patient outcomes following primary THA [64]. Given the risk and devastating sequelae that can follow intraoperative fractures, care must be taken to assess the stock during broaching and implant trials. We find that during our implementation of the DAA, direct visualization of the calcars and GT is paramount to prevent varus broaching and decrease intraoperative fracture risk. Care should also be exercised to properly lateralize the femur to prevent varus placement and to lessen the chance of intraoperative fractures. Additionally, careful examination of the soft tissue prior to and after any of these surgical approaches may aid in reducing fracture risk [64].

The DAA and its associated learning curve could increase the risk of intraoperative fracture in inexperienced hands. Cohen et al [66] compared the rate of intraoperative fractures using the DAA in 487 patients with and without the use of a fracture table. They found that the overall rate of intraoperative femur fracture (IFF) was 2.6% and was more likely to occur in patients greater than 70 years of age [66].

Aggarwal et al [67] recently reviewed complication rates between the DAA, posterior approach, direct lateral approach, and northern approach. Of the 30 intraoperative fractures that they identified in their data, ten (30%) occurred during the DAA, fourteen in the posterior group (46%), four in the northern group (13.3%), and two (0.67%) in the direct lateral group [67]. Although, after comparing the periprosthetic fracture rate in the DAA with the other approaches they found no statistically significant difference in intraoperative fracture rate between the approaches.

### Patient demand, perception, and marketing of the DAA

With the increasing popularity of the DAA, hospitals and major medical groups are attempting to capitalize on the popularity of the approach. A recent study by Shofoluwe et al [68] reviewed the number of AAHKS members who had mention or information regarding the DAA on their websites [68]. Their data indicated that roughly 20% of AAHKS members’ websites discussed the “advantages” of the DAA. Claims on the websites included that the DAA was less invasive/muscle sparring, decreased pain and risk of dislocation, shortened LOS, and led to quicker recovery. Interestingly, only 3.6% of the websites examined contained peer-reviewed referenced literature supporting the claims made. The responsibility to properly inform patients is paramount in medicine and should not be taken lightly especially in the cases of life-altering surgery. A patient’s knowledge of the DAA may be influenced by many factors. As mentioned earlier, Trousdale et al [53] examined where patients received the majority of their knowledge regarding the surgical approach before primary THA [53]. Although a majority of patients received their information via friends or family, 38% still received their information from a healthcare professional, including their websites and educational literature. Therefore, these claims about the DAA can unduly influence patient decisions and should be presented with caution on individual websites.

## Conclusion

The DAA to the hip has a complex history and is advocated by some over other surgical approaches in THA. However, to date, the published literature remains inconclusive on this ongoing debate. A debate between surgeons who believe their respective approach is superior remains unjustifiable as surgical experience, and comfortability with any approach may be the most important factor. The DAA is among many other surgical techniques for performing THA. The decision to employ a certain approach should be based on training and personal preference. Further long-term studies will aid in determining if the DAA with its contemporary resurgence is superior, equal, or inferior to other approaches to the hip.

## Data Availability

Not applicable.

## Abbreviations

DAA: Direct anterior approach
THA: Total hip arthroplasty
TFL: Tensor fasciae latae
LOS: Length of stay
AAHKS: American Association of Hip and Knee Surgeons
BMI: Body mass index
ASIS: Anterior superior iliac spine
GT: Greater trochanter
EBL: Estimated blood loss
PRO: Patient reported outcomes
PL: Posterolateral
FJS: Forgotten joint score
WOMAC: Western Ontario and McMaster Universities Arthritis Index
PASS: Patient Acceptable Symptomatic State
MCID: Minimal clinically important difference

## References

[1] Rachbauer F, Kain MSH, Leunig M. The history of the anterior approach to the hip. Orthop Clin North Am 2009;40:311–320. https://doi.org/10.1016/j.ocl.2009.02.007. 10.1016/j.ocl.2009.02.007 19576398

[2] Judet J, Judet H (1985). Voie d’abord anterieure dans l’arthroplastie totale de la hanche. Press Medicale.

[3] Smith-petersen MN, Larson CB (1947). Complications of old fractures of the neck of the femur; results of treatment of vitallium-mold arthroplasty. J Bone Joint Surg Am.

[4] Light TR, Keggi KJ. Anterior approach to hip arthroplasty. Clin Orthop Relat Res. 1980:255–60. 7438611

[5] Post ZD, Orozco F, Diaz-Ledezma C, Hozack WJ, Ong A (2014). Direct anterior approach for Total hip Arthroplasty. J Am Acad Orthop Surg.

[6] Kyriakopoulos G, Poultsides L, Christofilopoulos P (2018). Total hip arthroplasty through an anterior approach: the pros and cons. EFORT Open Rev.

[7] Restrepo C, Parvizi J, Pour AE, Hozack WJ (2010). Prospective randomized study of two surgical approaches for Total hip Arthroplasty. J Arthroplast.

[8] Thaler M, Dammerer D, Krismer M, Ban M, Lechner R, Nogler M (2019). Extension of the direct anterior approach for the treatment of Periprosthetic femoral fractures. J Arthroplast.

[9] Patel NN, Shah JA, Erens GA (2019). Current trends in clinical practice for the direct anterior approach Total hip Arthroplasty. J Arthroplast.

[10] Mast NH, Laude F (2011). Revision Total hip Arthroplasty performed through the Hueter interval. J Bone Jt Surg.

[11] Yue C, Kang P, Pei F. Comparison of direct anterior and lateral approaches in total hip arthroplasty: A systematic review and meta-analysis (PRISMA). Med (United States). 2015:94. 10.1097/MD.0000000000002126. 10.1097/MD.0000000000002126 PMC5058892 26683920

[12] Abdel MP, Berry DJ (2019). Current practice trends in primary hip and knee Arthroplasties among members of the American Association of hip and Knee Surgeons: A long-term update. J Arthroplast.

[13] Stone AH, Sibia US, Atkinson R, Turner TR, King PJ (2018). Evaluation of the learning curve when transitioning from Posterolateral to direct anterior hip Arthroplasty: A consecutive series of 1000 cases. J Arthroplast.

[14] Ponzio DY, Poultsides LA, Salvatore A, Lee Y, yu, Memtsoudis SG, Alexiades MM. (2018). In-hospital morbidity and postoperative revisions after direct anterior vs posterior Total hip Arthroplasty. J Arthroplast.

[15] Higgins BT, Barlow DR, Heagerty NE, Lin TJ (2015). Anterior vs. posterior approach for Total hip Arthroplasty, a systematic review and Meta-analysis. J Arthroplast.

[16] Kennon R, Keggi J, Zatorski LE, Keggi KJ (2004). Anterior approach for Total hip Arthroplasty. J Bone Jt Surg.

[17] Bs B, Vallurupalli S (2008). Minimally invasive total hip arthroplasty with the anterior approach. Indian J Orthop.

[18] Connolly KP, Kamath AF (2016). Direct anterior total hip arthroplasty: literature review of variations in surgical technique. World J Orthop.

[19] Bender B, Nogler M, Hozack WJ (2009). Direct anterior approach for Total hip Arthroplasty. Orthop Clin North Am.

[20] Moskal JT, Capps SG, Scanelli JA (2013). Anterior muscle sparing approach for total hip arthroplasty. World J Orthop.

[21] Barrett WP, Turner SE, Leopold JP (2013). Prospective randomized study of direct anterior vs postero-lateral approach for total hip arthroplasty. J Arthroplast.

[22] Sierra RJ, Mabry TM, Sems SA, Berry DJ (2013). Acetabular fractures: the role of total hip replacement. Bone Joint J.

[23] Sang W, Zhu L, Ma J, Lu H, Wang C (2016). The influence of body mass index and hip anatomy on direct anterior approach Total hip replacement. Med Princ Pract.

[24] Jahng KH, Bas MA, Rodriguez JA, Cooper HJ (2016). Risk factors for wound complications after direct anterior approach hip Arthroplasty. J Arthroplast.

[25] Sali E, Marmorat JL, Gaudot F, Nich C (2019). Perioperative complications and causes of 30- and 90-day readmission after direct anterior approach primary total hip arthroplasty. J Orthop.

[26] Galakatos GR (2018). Direct anterior Total hip Arthroplasty. Mo Med.

[27] Horne PH, Olson SA (2011). Direct anterior approach for total hip arthroplasty using the fracture table. Curr Rev Musculoskelet Med.

[28] Baba T, Homma Y, Jinnai Y, Tanabe H, Banno S, Watari T, et al. Posterior versus direct anterior approach in revision hip arthroplasty using Kerboull-type plate. SICOT-J. 2020:6. 10.1051/SICOTJ/2019040. 10.1051/sicotj/2019040 PMC6959137 31934846

[29] Nakamura J, Hagiwara S, Orita S, Akagi R, Suzuki T, Suzuki M (2017). Direct anterior approach for total hip arthroplasty with a novel mobile traction table -a prospective cohort study. BMC Musculoskelet Disord.

[30] Bechler U, Springer B, Boettner F. Anterior primary Total hip Arthroplasty. Total hip replace. - an Overv. InTech. 2018. 10.5772/intechopen.76070.

[31] Vandeputte FJ, Vanbiervliet J, Sarac C, Driesen R, Corten K (2021). Capsular resection versus capsular repair in direct anterior approach for total hip arthroplasty: a randomized controlled trial. Bone Joint J.

[32] Moretti VM, Post ZD. Surgical approaches for Total hip Arthroplasty. Indian J Orthop. n.d.;51:368–76. 10.4103/ortho.IJOrtho_317_16. 10.4103/ortho.IJOrtho_317_16 PMC5525517 28790465

[33] Zhao G, Zhu R, Jiang S, Xu N, Bao H, Wang Y. Using the anterior capsule of the hip joint to protect the tensor fascia lata muscle during direct anterior total hip arthroplasty: A randomized prospective trial. BMC Musculoskelet Disord. 2020;21. 10.1186/s12891-019-3035-9. 10.1186/s12891-019-3035-9 PMC6955089 31926554

[34] Bradley MP, Benson JR, Muir JM (2019). Accuracy of Acetabular component positioning using computer-assisted navigation in direct anterior Total hip Arthroplasty. Cureus.

[35] Carender CN, Meyer MD, Wynn MS, Bedard NA, Otero JE, Brown TS (2020). The prevalence of abnormal Spinopelvic relationships in patients presenting for primary Total hip Arthroplasty. Arthroplast Today.

[36] Haffer H, Adl Amini D, Perka C, Pumberger M (2020). The impact of Spinopelvic mobility on Arthroplasty: implications for hip and spine surgeons. J Clin Med.

[37] Boettner F, Zingg M, Emara AK, Waldstein W, Faschingbauer M, Kasparek MF (2017). The accuracy of Acetabular component position using a novel method to determine Anteversion. J Arthroplast.

[38] Dall ‘Oca C, Ceccato A, Cresceri M, Scaglia M, Guglielmini M, Pelizzari G (2020). Facing complications of direct anterior approach in total hip arthroplasty during the learning curve. Acta Biomed.

[39] Vles GF, Corten K, Driesen R, van Elst C, Ghijselings SG. Hidden blood loss in direct anterior total hip arthroplasty: a prospective, double blind, randomized controlled trial on topical versus intravenous tranexamic acid. Musculoskelet Surg. 2020. 10.1007/s12306-020-00652-0. 10.1007/s12306-020-00652-0 32152813

[40] Nairn L, Gyemi L, Gouveia K, Ekhtiari S, Khanna V. The learning curve for the direct anterior total hip arthroplasty: a systematic review. Int Orthop. 2021. 10.1007/s00264-021-04986-7. 10.1007/s00264-021-04986-7 33629172

[41] Pirruccio K, Evangelista PJ, Haw J, Goldberg T, Sheth NP (2020). Safely implementing the direct anterior Total hip Arthroplasty: A methodological approach to minimizing the learning curve. J Am Acad Orthop Surg.

[42] Garbarino L, Gold P, Sodhi N, Iturriaga C, Mont MA, Boraiah S (2021). Does structured postgraduate training affect the learning curve in direct anterior Total hip Arthroplasty? A single Surgeon’s first 200 cases. Arthroplast Today.

[43] Goytia RN, Jones LC, Hungerford MW (2012). Learning curve for the anterior approach total hip arthroplasty. J Surg Orthop Adv.

[44] de Steiger RN, Lorimer M, Solomon M (2015). What is the learning curve for the anterior approach for Total hip Arthroplasty?. Clin Orthop Relat Res.

[45] Zhao HY, De KP, Xia YY, Shi XJ, Nie Y, Pei FX (2017). Comparison of early functional recovery after Total hip Arthroplasty using a direct anterior or Posterolateral approach: A randomized controlled trial. J Arthroplast.

[46] Peters RM, van Beers LWAH, van Steenbergen LN, Wolkenfelt J, Ettema HB, ten Have BLEF (2018). Similar superior patient-reported outcome measures for anterior and Posterolateral approaches after Total hip Arthroplasty: postoperative patient-reported outcome measure improvement after 3 months in 12,774 primary Total hip Arthroplasties using the anterior, anterolateral, straight lateral, or Posterolateral approach. J Arthroplast.

[47] Taunton MJ, Mason JB, Odum SM, Springer BD (2014). Direct anterior total hip arthroplasty yields more rapid voluntary cessation of all walking aids: A prospective, randomized clinical trial. J Arthroplast.

[48] Singh V, Zak S, Schwarzkopf R, Davidovitch R (2020). Forgotten joint score in THA: comparing the direct anterior approach to posterior approach. J Arthroplast.

[49] Martusiewicz A, Delagrammaticas D, Harold RE, Bhatt S, Beal MD, Manning DW (2020). Anterior versus posterior approach total hip arthroplasty: patient-reported and functional outcomes in the early postoperative period. Hip Int.

[50] Charney M, Paxton EW, Stradiotto R, Lee JJ, Hinman AD, Sheth DS (2020). A comparison of risk of dislocation and cause-specific revision between direct anterior and posterior approach following elective Cementless Total hip Arthroplasty. J Arthroplast.

[51] Cheng TE, Wallis JA, Taylor NF, Holden CT, Marks P, Smith CL (2017). A prospective randomized clinical trial in Total hip Arthroplasty—comparing early results between the direct anterior approach and the posterior approach. J Arthroplast.

[52] Sauder N, Vestergaard V, Siddiqui S, Galea VP, Bragdon CR, Malchau H (2020). The AAHKS clinical research award: no evidence for superior patient-reported outcome scores after Total hip Arthroplasty with the direct anterior approach at 1.5 months postoperatively, and through a 5-year follow-up. J Arthroplast.

[53] Trousdale WH, Taunton MJ, Mabry TM, Abdel MP, Trousdale RT (2017). Patient perceptions of the direct anterior hip Arthroplasty. J Arthroplast.

[54] Maratt JD, Gagnier JJ, Butler PD, Hallstrom BR, Urquhart AG, Roberts KC (2016). No difference in dislocation seen in anterior Vs posterior approach Total hip Arthroplasty. J Arthroplast.

[55] Huerfano E, Bautista M, Huerfano M, Nossa JM. Use of Surgical Approach Is Not Associated With Instability After Primary Total Hip Arthroplasty: A Meta-analysis Comparing Direct Anterior and Posterolateral Approaches. J Am Acad Orthop Surg. 2020;Publish Ah. 10.5435/JAAOS-D-20-00861. 10.5435/JAAOS-D-20-00861 33315648

[56] Siljander MP, Whaley JD, Koueiter DM, Alsaleh M, Karadsheh MS (2020). Length of stay, discharge disposition, and 90-day complications and revisions following primary Total hip Arthroplasty: A comparison of the direct anterior, Posterolateral, and direct superior approaches. J Arthroplast.

[57] Pincus D, Jenkinson R, Paterson M, Leroux T, Ravi B (2020). Association between surgical approach and major surgical complications in patients undergoing Total hip Arthroplasty. JAMA.

[58] Matta JM, Shahrdar C, Ferguson T (2005). Single-incision anterior approach for total hip arthroplasty on an orthopaedic table. Clin Orthop Relat Res.

[59] Sariali E, Leonard P, Mamoudy P (2008). Dislocation after total hip arthroplasty using Hueter anterior approach. J Arthroplast.

[60] Siguier T, Siguier M, Brumpt B. Mini-incision anterior approach does not increase dislocation rate: a study of 1037 total hip replacements. Clin Orthop Relat Res. 2004;164–73. 10.1097/01.BLO.0000136651.21191.9F. 10.1097/01.blo.0000136651.21191.9f 15346069

[61] Tsukada S, Wakui M (2015). Lower dislocation rate following Total hip Arthroplasty via direct anterior approach than via posterior approach: five-year-average follow-up results. Open Orthop J.

[62] Demos HA, Rorabeck CH, Bourne RB, MacDonald SJ, McCalden RW. Instability in primary total hip arthroplasty with the direct lateral approach. Clin Orthop Relat Res. 2001:168–80. 10.1097/00003086-200112000-00020. 10.1097/00003086-200112000-00020 11764347

[63] Masonis JL, Bourne RB. Surgical approach, abductor function, and total hip arthroplasty dislocation. Clin Orthop Relat Res. 2002:46–53. 10.1097/00003086-200212000-00006. 10.1097/00003086-200212000-00006 12461355

[64] Petis S, Howard JL, Lanting BL, Vasarhelyi EM (2015). Surgical approach in primary total hip arthroplasty: anatomy, technique and clinical outcomes. Can J Surg.

[65] Farrell CM, Springer BD, Haidukewych GJ, Morrey BF (2005). Motor nerve palsy following primary total hip arthroplasty. J Bone Joint Surg Am.

[66] Cohen EM, Vaughn JJ, Ritterman SA, Eisenson DL, Rubin LE (2017). Intraoperative femur fracture risk during primary direct anterior approach Cementless Total hip Arthroplasty with and without a fracture table. J Arthroplast.

[67] Aggarwal VK, Elbuluk A, Dundon J, Herrero C, Hernandez C, Vigdorchik JM, et al. Surgical approach significantly affects the complication rates associated with total hip arthroplasty. Bone Jt J. 2019. 10.1302/0301-620X.101B6.BJJ-2018-1474.R1. 10.1302/0301-620X.101B6.BJJ-2018-1474.R1 31154834

[68] Shofoluwe AI, Naveen NB, Inabathula A, Ziemba-Davis M, Meneghini RM, Callaghan JJ (2018). Internet promotion of direct anterior approach Total hip Arthroplasty by members of the American Association of hip and Knee Surgeons. J Arthroplast.

